# Pre-diabetes is associated with altered functional connectivity density in cortical regions of the default-mode network

**DOI:** 10.3389/fnagi.2022.1034355

**Published:** 2022-11-10

**Authors:** Karel M. Lopez-Vilaret, Marina Fernandez-Alvarez, Ehsan Shokri-Kojori, Dardo Tomasi, Jose L. Cantero, Mercedes Atienza

**Affiliations:** ^1^Laboratory of Functional Neuroscience, Pablo de Olavide University, Seville, Spain; ^2^CIBERNED, Network Center for Biomedical Research in Neurodegenerative Diseases, Madrid, Spain; ^3^National Institute on Alcohol Abuse and Alcoholism, Bethesda, MD, United States

**Keywords:** pre-diabetes, insulin resistance, brain functional connectivity, functional connectivity density mapping, cognition

## Abstract

Insulin resistance and glucose dysregulation are associated with patterns of regional brain hypometabolism characteristic of Alzheimer’s disease (AD). As predicted by evidence linking brain glucose metabolism to brain functional connectivity, type 2 diabetes is accompanied by altered functional connectivity density (FCD) in regions highly vulnerable to AD, but whether these alterations start at earlier stages such as pre-diabetes remain to be elucidated. Here, in addition to assessing whether pre-diabetes leads to a functional reorganization of densely connected cortical areas (hubs), we will assess whether such reorganization is conditioned by sex and/or insulin resistance, and contributes to improved cognition. One hundred and forty-four cognitively unimpaired middle-aged and older adults (55–78 years, 79 females), 73 with normoglycemia and 71 with pre-diabetes, underwent resting-state fMRI scanning. We first computed FCD mapping on cortical surfaces to determine the number of short- and long-range functional connections of every vertex in the cortex, and next used hubs showing aberrant FCD as seeds for the resting-state functional connectivity (rs-FC) calculation. ANCOVAs and linear multiple regression analyses adjusted by demographic and cardiometabolic confounders using frequentist and Bayesian approaches were applied. Analyses revealed higher long-range FCD in the right precuneus of pre-diabetic females and lower short-range FCD in the left medial orbitofrontal cortex (mOFC) of pre-diabetic individuals with higher insulin resistance. Although the mOFC also showed altered rs-FC patterns with other regions of the default mode network in pre-diabetic individuals, it was FCD of the precuneus and mOFC, and not the magnitude of their rs-FC, that was associated with better planning abilities and Mini-Mental State Examination (MMSE) scores. Results suggest that being female and/or having high insulin resistance exacerbate pre-diabetes-induced alterations in the FCD of hubs of the default-mode network that are particularly vulnerable to AD pathology. These changes in brain network organization appear to be compensatory for pre-diabetic females, likely assisting them to maintain cognitive functioning at early stages of glucose dysregulation.

## Introduction

Pre-diabetes is a chronic metabolic condition in which plasma glucose concentrations exceed normal levels without reaching the diabetes thresholds, while increasing the risk for the development of type 2 diabetes (Tabák et al., 2012). Like diabetes (Luchsinger et al., 2007), pre-diabetes also increases the risk for cognitive decline (Marseglia et al., 2019) and AD (Garfield et al., 2021), probably through altered cerebral metabolic pathways. Accordingly, both pre-diabetes and insulin resistance, the main determinant of pre-diabetes (Cai et al., 2019), have been associated with AD-like patterns of brain hypometabolism in older adults (Baker et al., 2011; Sundermann et al., 2021). Interestingly, this association seems to be moderated by sex when cognitive impairment becomes evident (Sundermann et al., 2021). Thus, pre-diabetic women with mild cognitive impairment (MCI) not only show an earlier dementia onset, but also exhibit a more noticeable hypometabolism of posterior cingulate, angular, and middle/inferior temporal gyri than pre-diabetic men (Sundermann et al., 2021), likely as a result of the metabolic changes associated with the loss of female sex hormones after menopause (Demetrius et al., 2021). Although it is unknown whether this sexual dimorphism is also evident in diabetic patients with or without MCI, a recent animal study has found that diabetes-induced brain hypometabolism is exacerbated in female rats (Wu et al., 2021).

As predicted by evidence linking brain glucose metabolism to brain functional connectivity (Tomasi et al., 2013; Riedl et al., 2014), pre-diabetes and diabetes have been associated with disturbances in local and global brain network organization (van Bussel et al., 2016; Liu et al., 2018; Li et al., 2020; Feng et al., 2021). These anomalies have been identified by applying data-driven analysis strategies to resting-state fMRI data. These approaches are based on brain-wide correlation of blood-oxygenation level dependent (BOLD) time series between voxels without the need of selecting a region of interest to act as a seed. They include graph theoretical network analysis, which estimates the brain’s ability to globally integrate information while maintaining functional specialization, and FCD mapping, which identifies specific brain regions displaying a high degree of short- and long-range functional connections, representing brain hubs. These methods have shown that pre-diabetes is characterized by abnormally high local efficiency that worsens with progression to diabetes, presumably to compensate for cognitive decline (van Bussel et al., 2016). Diabetes has also been associated with increased degree centrality (i.e., global connectivity) in the right cerebellum (Li et al., 2020) and regions of the temporal lobe and hippocampus (Feng et al., 2021), decreased long-range FCD in regions of the visual network, and increased short-range FCD in nodes of the default mode network and fronto-parietal network (Liu et al., 2018), part of which were related to impaired executive function (Liu et al., 2018; Feng et al., 2021). Interestingly, abnormal FCD patterns have also been reported in some of these hubs in patients with MCI and AD that correlated positively with MMSE scores (Mao et al., 2021). However, whether pre-diabetes is also associated with disrupted balance of long- and short-range FCD in densely connected hubs highly vulnerable to AD remains to be elucidated. This knowledge would provide a therapeutic target to work on in order to slow or delay the cognitive decline associated with this metabolic condition.

Here, we first aim to identify cortical FCD hubs that are altered in pre-diabetic individuals by applying FCD mapping at the surface level and then determine whether their number of functional connections and magnitude of rs-FC with other cortical regions (also at the surface level) is conditioned by sex and/or insulin resistance. We applied the surface-based approach because it contributes to increase the specificity of connectivity results by reducing signal contamination between neighboring functional brain regions (Brodoehl et al., 2020). Given the wide variety of female-biased changes during aging that contribute specifically to the development of AD (Uchoa et al., 2016; Pike, 2017; Guo et al., 2022) and the role of insulin resistance as a common link between diabetes and AD (Ferreira et al., 2018; Folch et al., 2019), the association between pre-diabetes and altered FCD is expected to be stronger in females and in individuals with higher insulin resistance, especially in cortical hubs that are particularly vulnerable to AD pathology at early stages of the disease, like the precuneus, posterior cingulate and medial orbitofrontal cortices (Palmqvist et al., 2017). On the other hand, pre-diabetes has been associated with a progressive decline in executive function in women with MCI (Sundermann et al., 2021), so if there is any relationship between abnormal FCD and cognition at the cross-sectional level, we expect it to be with this cognitive domain, and that such an association would be specific to women. If we also consider the evidence linking pre-diabetes to increased functional specialization, presumably in order to prevent cognitive decline (van Bussel et al., 2016), we hypothesized that alterations in FCD, mainly at the local level (i.e. short-range FCD), will contribute to improved cognition, especially in women.

## Materials and methods

### Participants

One hundred and forty-four cognitively unimpaired middle and older adults (55–78 years, 67.6 ± 4.8 years, 79 females), 73 with normoglycemia and 71 with pre-diabetes [i.e., fasting blood glucose of 5.6–6.9 mmol/l (American Diabetes Association, 2014)] participated in the study. All of them underwent neurological and neuropsychological assessment to discard both the presence of dementia and objective cognitive impairment. Participants met the following criteria: (i) normal cognitive performance in the neuropsychological tests relative to appropriate reference values for age and education level; (ii) global score of 0 (no dementia) in the Clinical Dementia Rating; (iii) functional independence as assessed by the Spanish version of the Interview for Deterioration in Daily Living Activities (Böhm et al., 1998); and (iv) scores ≤ 5 (no depression) in the short form of the Geriatric Depression Scale (Sheikh and Yesavage, 1986). Individuals with severe visual and/or hearing loss, contraindications to MRI, having been diagnosed with or taking medication for diabetes, showing fasting blood glucose > 6.9 mmol/l, taking medication that may affect cognition, and/or with medical conditions that affect brain structure or function (e.g., cerebrovascular disease, epilepsy, head trauma, history of neurodevelopmental disease, alcohol abuse, hydrocephalus, and/or intracranial mass) were not included in the study. All participants gave informed consent to the experimental protocol approved by the Ethical Committee for Clinical Research of the Junta de Andalucía according to the principles outlined in the Declaration of Helsinki.

### Cognitive assessment

Neuropsychological assessment included MMSE as a measure of global cognitive function and specific tests to measure different aspects of executive function like the semantic and letter verbal fluency tests based on the “Animal” and letter “P” naming tasks, the two forms of the Trail Making Test (TMT-A and TMT-B), the Tower of London (ToL), and the Spatial Span subtest of the Wechsler Memory Scale-III (WMS-III). The test scores were z-transformed and multiplied by (−1) when greater scores were indicative of poorer performance.

### Biochemical measurements

Blood samples were taken in the morning, after 12 h of fasting. Serum levels of glucose, total cholesterol, LDL, HDL, and triglycerides were obtained with the automated A15 Random Access Analyzer^®^ (Biosystems, Barcelona, Spain) using Biosystems reagents. Serum insulin levels were determined with Quantikine ELISA kits (R&D Systems, Minneapolis, MN, USA). Adiponectin and leptin were measured in plasma with Luminex human bead-based assays (Bio-Techne R&D Systems).

### Estimation of insulin resistance, cardiorespiratory fitness, and metabolic status

The insulin resistance was estimated with the homeostatic model assessment (HOMA) score by dividing the product of fasting serum insulin (mU/l) and glucose (mmol/l) by 22.5 (Matthews et al., 1985). SI units for insulin were transformed to conventional units by dividing by a factor of 6 (Knopp et al., 2019).

All models were adjusted by cardiorespiratory fitness (CRF) and metabolic status (MS), two well-established cardiometabolic risk factors for AD (Tari et al., 2019; Kim et al., 2021). In addition, higher CRF has been associated with better executive function through changes in functional brain connectivity (Peven et al., 2019), while the metabolic syndrome has been linked to poorer executive function presumably as a result of altered intrinsic communication across core neural networks and disrupted between-network connections across the brain (Foret et al., 2021; Rashid et al., 2021).

Cardiorespiratory fitness was estimated from an equation validated in 1,458 men and 405 women aged 20 to 70 years (Jurca et al., 2005). The equation includes sex (0 for women and 1 for men), age, body mass index (BMI), resting heart rate, and self-reported physical activity (SRPA) from a questionnaire asking participants to identify the physical activity category out of 5 that best reflects their daily physical activity pattern. The CRF, expressed in ml/min/kg, was computed as follows:


C⁢R⁢F=S⁢e⁢x× 2.77-A⁢g⁢e× 0.1-B⁢M⁢I× 0.17

-H⁢e⁢a⁢r⁢t⁢r⁢a⁢t⁢e× 0.04+S⁢R⁢P⁢A+18.07

The MS was defined as a continuous score that is not specific to that sample of participants (Soldatovic et al., 2016) as follows:


$$M\,S=2\times \frac{W\,a\,i\,s\,t}{H\,e\,i\,g\,h\,t}+\frac{G\,l\,u\,c\,o\,s\,e}{5.6\,m\,m\,o\,l/l}+\frac{T\,r\,i\,g\,l\,y\,c\,e\,r\,i\,d\,e\,s}{1.7\,m\,m\,o\,l/l}$$

+S⁢y⁢s⁢t⁢o⁢l⁢i⁢c⁢p⁢r⁢e⁢s⁢s⁢u⁢r⁢e130⁢m⁢m⁢H⁢g-H⁢D⁢L40⁢(m⁢e⁢n)⁢o⁢r⁢ 50⁢(w⁢o⁢m⁢e⁢n)

### Magnetic resonance imaging acquisition

Structural and functional brain images were acquired on a 3T Philips Ingenia MRI scanner equipped with a 32-channel head coil for reception (Philips, Best, Netherlands) at Pablo de Olavide University. The session started with a 3D T1-weighted (T1w) magnetization prepared rapid gradient echo (MPRAGE) sequence acquired in the sagittal plane: repetition time = 2,600 ms, echo time = 4.7 ms, flip angle (FA) = 9°, matrix = 384 × 384, voxel resolution = 0.65 mm^3^ isotropic, and no gap between slices. Next, T2w Fast Field Echo images were acquired using a BOLD sensitive single-shot echo-planar imaging (EPI) sequence in the axial plane during resting state: repetition time/echo time: 2,000/30 ms, FA = 80°, matrix = 80 × 80 mm, voxel resolution = 3 mm^3^ isotropic, resulting in 35 slices with 1 mm of gap between adjacent slices. We acquired fMRI time series with 250 EPI volumes to achieve stable functional connectivity metrics (Tomasi et al., 2017), preceded by 4 dummy volumes. Pulse and respiratory signals were recorded for retrospective correction of physiological artifacts of extracerebral origin.

### Magnetic resonance imaging data preprocessing

Structural MRI data was processed using the pipeline of Freesurfer v6.0.^1^ Pial surface misplacements and erroneous white matter segmentation was manually corrected on a slice-by-slice basis.

The resting-state fMRI data were preprocessed in the subject-space using functions of the AFNI software (AFNI_20.3.01).^2^ For each participant, high-frequency spikes were eliminated (*3dDespike*), time-locked cardiac and respiratory motion artifacts on cerebral BOLD signals were minimized using RETROICOR (Glover et al., 2000), time differences in slice-acquisition were corrected (*3dTshift*), EPI scans were aligned using rigid body motion correction with the first volume as reference (*3dVolreg*), and aligned EPI scans were co-registered to their corresponding T1w volumes (*align_epi_anat.py;* cost function: lpc + ZZ).

Outlier volumes were removed if more than 5% of the voxels showed a signal with an intensity higher than the median absolute deviation of the time series (*3dToutcount*) and/or if they exceeded head motion > 0.3 mm (*1d_tool.py -censor_motion*). None of the participants exceeded 20% of artifactual volumes after volume censoring. Nuisance regression (*3dTproject*) was applied to remove linear drifts and minimize the impact of non-neuronal fluctuations on fMRI signal by including: (i) six head motion parameters with their first-order derivatives, (ii) time series of mean total white matter/cerebrospinal fluid signal intensity, and (iii) pulse/respiratory fluctuations plus their derivatives to mitigate effects of extracerebral physiological noise. No temporal band-pass filtering was applied.

### Functional connectivity density mapping

Whole-cortex, surface-based FCD mapping was computed using custom MATLAB scripts (The MathWorks, Natick, MA, USA) (Tomasi and Volkow, 2010). First, we calculated global FCD for each cortical vertex by computing the number of functional connections with the remaining vertices that exceed the Pearson’s correlation R > 0.6. Next, we computed short-range FCD (srFCD) whose magnitude depends on how many local functional connections maintain each vertex with its neighbors. For this purpose, a growing algorithm was applied by which a vertex (*x*_j_) was added to the list of neighbors (*x*_0_) if it was adjacent to a vertex that was linked to *x*_0_ by a continuous path of functionally connected vertices, and the correlation coefficient between *x*_0_ and *x*_j_ was > 0.6. This calculation was iteratively repeated for all vertices that were adjacent to the neighbors of *x*_0_ until no new neighbors could be added to the list. The long-range FCD (lrFCD) was computed by subtracting the srFCD from the global FCD. Finally, srFCD and lrFCD cortical maps were transformed to z scores and spatially smoothed using a Gaussian kernel with full width at half maximum (FWHM) of 6 mm.

### Seed-to-whole cortex resting-state functional connectivity

Cortical hubs showing FCD significantly associated with pre-diabetes, moderated or not by sex and/or HOMA, were used as seeds in subsequent rs-FC analysis. For each participant, the mean BOLD time series were extracted from each significant cluster and correlated to the time series of all of vertices within the gray matter mask. The resulting rs-FC maps were Fisher’s z transformed and spatially smoothed (FWHM = 6 mm). We next performed a one-sample-*t*-test in each group to identify clusters showing significant positive rs-FC. By combining the binary spatial maps of the two groups, we obtained a positive spatial mask where subsequent surface-based statistical analyses were performed. In cases where the seed corresponded to a dropout region such as the orbitofrontal cortex or inferior temporal gyrus (Ojemann et al., 1997), we assessed whether there was a main effect of group and/or group × sex or group × HOMA interaction effect on BOLD signal intensity.

### Statistical analysis

The main effect of group (normoglycemia vs. pre-diabetes) and group × sex interaction was assessed for demographic, anthropometric, cardiometabolic, and cognitive measures by applying two-sample *t*-tests and ANOVAs with the frequentist and Bayesian approach in R (R Core Team, 2020) and JASP, version 0.12.2,^3^ respectively. Bayesian methods overcome the problem of multiple comparisons.

One-way ANCOVAs based on the frequentist approach were first conducted with SurfStat^4^ to analyze the main effect of group on surface-based FCD and rs-FC patterns of cortical hubs after adjusting for age, sex, education years, BMI, HOMA, adiponectin/leptin (A/L) ratio, MS status, and CRF. The A/L ratio was included as a covariate of no interest because of its neuroprotective action under conditions of high insulin resistance and normoglycemia (Lopez-Vilaret et al., 2021).

To determine whether these associations were moderated by sex, we performed two-way ANCOVAs including the same covariates of no interest with the exception of sex. Next, we conducted linear multiple regression analyses to assess whether the association of pre-diabetes with FCD and/or rs-FC was moderated by HOMA and/or by the interaction between HOMA and sex. As pre-diabetes and HOMA are variables typically correlated, we made sure that the variance inflation factor was below 10 (Vittinghoff et al., 2012).

Multiple comparisons were corrected *via* a hierarchical statistical model that first controls the family-wise error (FEW) rate at the level of clusters by applying random field theory over smoothed statistical maps (p_verte_ < 0.001, p_cluster_ < 0.05), and next controls the false discovery rate at the level of vertex within each cluster (*p* < 0.05) over unsmoothed statistical values (Bernal-Rusiel et al., 2010). The location of each cluster’s peak vertex was identified on the Desikan-Killiany atlas (Desikan et al., 2006). The peak vertex of each cluster was next obtained for each participant to estimate the standardized effect size by applying the Cohen’s *d* (Cohen, 1992) for the main effect of group and group × sex interaction, and the standardized effect size (δ) for the group × HOMA interaction (Bodner, 2017). The precision of effect sizes was estimated by computing the bias-corrected and accelerated bootstrap 95% confidence intervals (CI_0.95_) through the function *bootci* implemented in MATLAB.

Next, we used the mean of the significant cluster of FCD and rs-FC to estimate the magnitude of the evidence in favor of the alternative hypothesis by applying the Bayesian approach. Bayesian analyses were based on non-informative priors such as the Jeffreys-Zellner-Siow prior with an r scale of 0.354 (Liang et al., 2008). We compared the strength of the Bayes factor for the model including all covariates of no interest (null model) with the model including the predictor of interest (experimental model) (BF_10_). We used the classification scheme proposed by Lee and Wagenmakers (2013) to interpret the BF_10_. The alternative hypothesis was considered valid if the contrast was significant with the frequentist approach (*p* < 0.05), the magnitude of the evidence in favor of this hypothesis was at least moderate with the Bayesian approach (BF_10_ ≥ 3), and the standardized effect size, although small, was significant (CI_0.95_ did not include zero).

Finally, we assessed whether the main and interaction effects found on the different clusters in FCD and rs-FC were associated with scores on neuropsychological tests using both frequentist and Bayesian approaches. The Yeo-Johnson transformation was applied to scores derived from the different neuropsychological tests to reduce the detrimental effects of skewedness and heteroscedasticity in the different models (Yeo and Johnson, 2000). We then assessed whether the mean of the significant cluster of FCD or rs-FC was associated with cognition as a function of group, or group and sex, or group and HOMA after adjusting for all other covariates of no interest. The same criteria mentioned above were applied to consider the alternative hypothesis as valid.

## Results

### Demographic, anthropometric, cardiometabolic, and cognitive measures

Table 1 shows the mean ± SD of demographic, anthropometric, cardiometabolic, and cognitive measures for the normoglycemia and pre-diabetes groups stratified by sex. As expected, the pre-diabetic group showed greater BMI (*p* = 0.0004, BF_10_ = 3.7), central obesity (*p* = 0.0001, BF_10_ = 327), and HOMA (*p* < 10^–7^, BF_10_ > 500) as well as worse MS status (*p* = 0.0001, BF_10_ = 192). We also found a significant group × sex interaction for triglycerides [*F*(1,140) = 8.3, *p* = 0.005, BF_10_ = 8.6], due to higher levels in pre-diabetic than normoglycemic females (*p* = 0.00006). There was no group difference or sex interaction for any of the cognitive scores (Table 1).

**TABLE 1:** Demographic, anthropometric, cardiometabolic, and cognitive measures for normoglycemic and pre-diabetic individuals stratified by sex.

Measures	Normoglycemia		Pre-diabetes		Statistical contrast	
	Females	Males	Females	Males	*Group*	*Group × Sex*
	(*n* = 39)	(*n* = 34)	(*n* = 40)	(*n* = 31)	*p*/BF_10_	*p*/BF_10_
***Demographic***						
Age (years)	66.9 ± 5.7	68.3 ± 4.9	66.9 ± 4.4	68.4 ± 4.0	0.99/0.18	0.97/0.24
Education (years)	10.1 ± 5.3	9.2 ± 5.2	10.2 ± 5.1	10.8 ± 4.6	0.38/0.25	0.37/0.33
***Anthropometric***						
BMI (kg/m^2^)	26.2 ± 3.5	26.1 ± 3.0	27.9 ± 3.2	28.8 ± 4.3	0.0004/3.7*	0.42/0.30
Waist (cm)	86.2 ± 9.6	89.5 ± 8.6	92.5 ± 9.7	97.1 ± 10.9	0.0001/327*	0.68/0.26
***Metabolic***						
Adiponectin (ng/ml)	6.5 ± 2.7	7.7 ± 2.2	6.2 ± 1.7	7.6 ± 1.8	0.52/0.22	0.67/0.26
Leptin (ng/ml)	22.7 ± 14.4	13.2 ± 15.7	24.4 ± 16.4	10.6 ± 8.9	0.99/0.18	0.37/0.34
A/L ratio (ng/ml)	0.4 ± 0.4	1.0 ± 0.6	0.4 ± 0.3	1.3 ± 1.3	0.44/0.24	0.08/0.88
LDL (mmol/l)	3.4 ± 0.8	3.1 ± 0.9	3.4 ± 0.8	3.1 ± 0.7	0.79/0.18	0.70/0.26
HDL (mmol/l)	1.5 ± 0.3	1.5 ± 0.4	1.6 ± 0.5	1.5 ± 0.6	0.97/0.18	0.76/0.26
Triglycerides (mmol/l)	1.0 ± 0.4	1.3 ± 0.6	1.6 ± 0.9	1.3 ± 0.7	0.0007/38.2*	0.005/8.6*
Glucose (mmol/l)	5.0 ± 0.3	5.0 ± 0.4	6.1 ± 0.4	6.1 ± 0.4	10^–15^ />500*	0.83/0.25
Insulin (pmol/l)	6.6 ± 2.7	9.2 ± 7.6	11.4 ± 5.4	10.4 ± 4.3	0.0006/43.8*	0.05/1.4
HOMA	1.5 ± 0.6	2.0 ± 1.5	3.1 ± 1.5	2.8 ± 1.2	10^–7^/>500*	0.06/1.2
MS status	2.3 ± 0.6	2.49 ± 0.6	2.93 ± 0.8	2.77 ± 0.8	0.0001/192*	0.13/0.58
***Cardiorespiratory***						
Heart rate (ppm)	64.5 ± 9.3	64.1 ± 11.4	68.7 ± 10.4	66.4 ± 10.9	0.05/1.0	0.57/0.28
SBP (mmHg)	129.2 ± 21.3	130.3 ± 24.2	133.4 ± 19.3	139.3 ± 17.3	0.07/0.82	0.48/0.31
CRF (ml/min/Kg)	7.5 ± 2.0	9.5 ± 1.8	7.5 ± 1.8	9.8 ± 1.8	0.89/0.18	0.62/0.25
***Cognitive***						
Phonetic fluency	13.6 ± 5.1	15.5 ± 4.7	15.3 ± 4.5	15.9 ± 5.0	0.18/0.41	0.39/0.33
Semantic fluency	20.1 ± 6.6	22.2 ± 5.2	23.3 ± 22.3	21.0 ± 6.1	0.57/0.21	0.31/0.37
TMT-A (sec)	45.8 ± 21.9	39.9 ± 14.2	41.3 ± 14.3	34.4 ± 10.9	0.08/0.72	0.85/0.22
TMT-B (sec)	122.2 ± 63.7	103.5 ± 48.1	132.0 ± 82.4	89.3 ± 41.1	0.99/0.18	0.25/0.43
Tower of London (sec)	379.8 ± 140.0	348.8 ± 136.5	409.6 ± 165.7	371.1 ± 134.4	0.26/0.32	0.88/0.26
**Spatial Span (WMS-III)**						
Forward	7.1 ± 1.7	8.1 ± 1.6	7.3 ± 1.6	7.3 ± 1.7	0.34/0.27	0.11/0.72
Backward	6.0 ± 2.1	6.4 ± 1.8	6.22 ± 1.7	6.6 ± 1.5	0.59/0.20	0.92/0.25
Total	13.1 ± 3.1	14.6 ± 3.0	13.5 ± 2.9	13.9 ± 2.7	0.77/0.19	0.30/0.42

All variables are expressed as mean ± SD. A/L, adiponectin/leptin; SBP, systolic blood pressure. *Rejection of the null hypothesis. Main effect of group and sex interaction.

### Functional connectivity density

**FIGURE 1:**
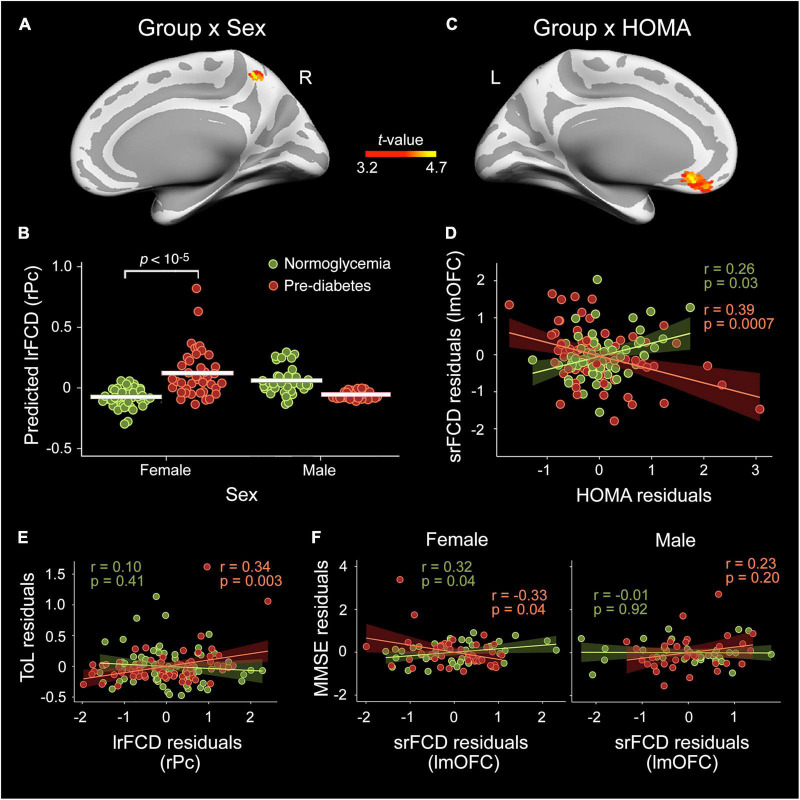
Moderating role of sex and homeostatic model assessment (HOMA) in the pre-diabetes-related alterations in functional connectivity density (FCD) cortical hubs. Effect of group × sex interaction in the long-range FCD (lrFCD) of the right precuneus (rPc) **(A,B)** and in the short-range FCD (srFCD) of the left medial orbitofrontal cortex (lmOFC) **(C,D)** after adjustment by age, sex, years of education, BMI, A/L ratio, CRF, and MS status. The shaded areas in the scatter plot reflect the confidence intervals (95%) for the fitted values. **(E)** Scatter plot illustrating the interaction between group and lrFCD in the rPC on the Tower of London (ToL) test scores. **(F)** Scatter plots illustrating the interaction between group, sex and srFCD in the lmOFC on Mini-Mental State Examination (MMSE) scores.

Table 2 and Figure 1 show results derived from whole-cortex surface-based FCD analyses. ANCOVAs revealed no group differences for either srFCD or lrFCD, but did reveal a group × sex interaction for lrFCD in the right precuneus (Figure 1A) [*F*(31,112) = 21.9, p_FWE_ = 0.03, BF_10_ = 165], due to higher values in pre-diabetic than in normoglycemic females (Figure 1B).

**TABLE 2:** Significant main effect of group and sex and homeostatic model assessment (HOMA) interaction on functional connectivity density (FCD) and resting-state functional connectivity (rs-FC).

*Connectivity measure: Contrast*	MNI coordinates	*R* ^2^	*F*(31,112)	*d*/δ	CI _95%_	BF_10_
Cortical location of the peak vertex						
***lrFCD: Group × Sex***						
R precuneus (*p* = 0.03)	7–47 55	0.13	21.9	−1.37^M^	−1.8–−0.09	165^E^
***srFCD: Group × HOMA-IR***						
L mOFC (*p* = 0.006)	−7 29–12	0.13	22.2	−1.14^M^	−2.2–−0.04	124^E^
***rs-FC L mOFC: Group***						
L PCC (*p* = 0.01)	−2–18 31	0.15	26	0.47^S^	0.16–0.77	49.7^VS^
R Parahipocampal (*p* = 0.007)	29–43–8	0.12	19.5	−0.50^S^	−0.80–−0.20	12.5^S^
***rs-FC L mOFC: Group × Sex***						
R IFG (pars opercularis) (*p* < 10^–6^)	44 10 5	0.13	22.2	−1.29^M^	−1.97–−0.61	353^E^
R STG (*p* = 0.008)	64–24 2	0.15	25.8	−0.99^S^	−1.55–−0.44	15.6^S^

L, left; R, right; mOFC, medial orbitofrontal cortex; PCC, posterior cingulate cortex; IFG, inferior frontal gyrus; STG, superior temporal gyrus. BF_10_: magnitude of the evidence in favor of the alternative hypothesis (^*E*^extreme, ^*VS*^very strong, ^*S*^strong, ^*M*^moderate).

Multiple linear regression on srFCD showed a pre-diabetes × HOMA interaction in the left mOFC (Figure 1C) [*F*(31,112) = 22.2, p_FWE_ = 0.006, BF_10_ = 124]. Particularly, the srFCD of the left mOFC decreased with increasing HOMA across pre-diabetic individuals (Figure 1D). Although this cortical region usually shows dropout, no main effect of group or group × sex/HOMA interaction were found for BOLD signal intensity in this region.

### Relationship between functional connectivity density and cognition

Regression analysis yielded a significant moderation effect of group on the association of lrFCD in the right precuneus with scores in the ToL test [*R*^2^ = 0.24, *F*(11,132) = 3.8, *p* = 0.0001, BF_10_ = 6.7]. *Post-hoc* analyses indicated that the higher the lrFCD in the right precuneus the better the performance in the ToL test in pre-diabetic individuals (Figure 1E). Additionally, the association between srFCD in the left mOFC and MMSE scores was moderated by group and sex [*R*^2^ = 0.17, *F*(14,129) = 2.0, *p* = 0.03, BF_10_ = 3.3]. *Post-hoc* analyses revealed increased MMSE scores with decreasing srFCD in the lmOFC in pre-diabetic females and the opposite relationship in normoglycemic females (Figure 1F).

### The resting-state functional connectivity of altered functional connectivity density hubs and its relationship with cognition

**FIGURE 2:**
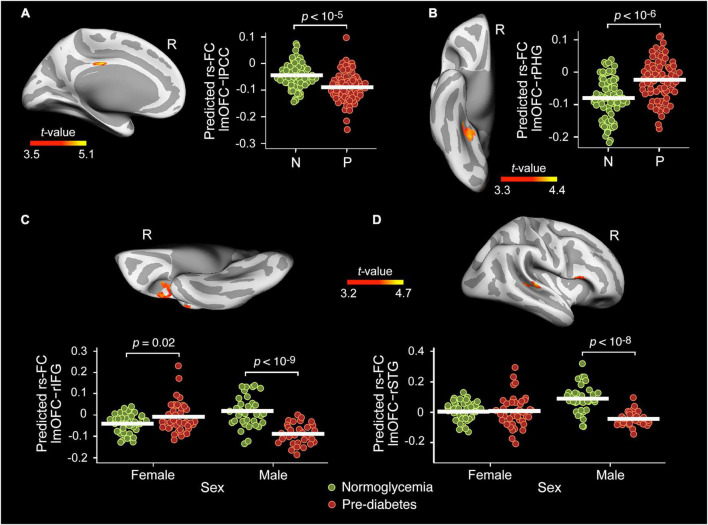
Main effect of group and sex interaction on the resting-state functional connectivity (rs-FC) of the lmOFC. Main effect of group in the rs-FC of the left medial orbitofrontal cortex (lmOFC) with the left posterior cingulate cortex (lPCC) **(A)** and right parahipocampal gyrus (rPHG) **(B)** adjusted by age, sex, years of education, BMI, A/L ratio, HOMA, CRF, and MS status. Effect of group × sex interaction in the rs-FC of the lmOFC with the right inferior frontal gyrus (rIFG) **(C)** and right superior temporal gyrus (rSTG) **(D)** adjusted by age, years of education, BMI, A/L ratio, HOMA, CRF, and MS status. N, Normoglycemia; P, Pre-diabetes.

Table 2 contains detailed information about the main effect of group and sex interaction on the rs-FC between the left mOFC and the whole cortex. No significant effect was found for the right precuneus. The ANCOVA showed group differences in the rs-FC of the lmOFC with the left posterior cingulate cortex (lPCC) [*F*(31,112) = 26.0, p_FWE_ = 0.01, BF_10_ = 49.7] and right parahipocampal gyrus (rPHG) [*F*(31,112) = 19.5, p_FWE_ = 0.007, BF_10_ = 12.5]. Compared to normoglycemia, the pre-diabetes group showed lower rs-FC with lPCC (Figure 2A) and higher rs-FC with rPHG (Figure 2B).

As indicated in Table 2, the ANCOVA also showed a group × sex interaction in the rs-FC of the lmOFC with the right inferior frontal gyrus (rIFG) [*F*(31,112) = 22.2, p_FWE_ < 10^–6^, BF_10_ = 353] and right superior temporal gyrus (rSTG) [*F*(31,112) = 25.8, p_FWE_ = 0.008, BF_10_ = 15.6]. The interaction effect was mainly due to the lower rs-FC shown by pre-diabetic males when compared with their normoglycemic counterparts (Figures 2C,D).

None of these abnormal rs-FC patterns were related to scores derived from cognitive testing employed in the present study. The Bayesian approach also revealed no evidence in favor of either the null or the alternative hypothesis.

## Discussion

The present study showed altered short- and long-range FCD in pre-diabetic females and pre-diabetic individuals with higher insulin resistance values after adjusting by demographic, anthropometric, metabolic, and cardiorespiratory measures. In particular, we identified increased lrFCD in the right precuneus of pre-diabetic females, and decreased srFCD in the left mOFC of pre-diabetic individuals the higher the insulin resistance. Contrary to the precuneus, FC of the mOFC was modulated by glycemic status and sex. However, it was the FCD of precuneus and mOFC, and not the magnitude of their rs-FC with other cortical regions, that correlated with planning abilities and global cognition in pre-diabetic females.

Previous studies have shown sex differences related to cognitive decline and AD in both pre-diabetes and diabetes. Diabetic females experience not only faster cognitive decline compared to their male counterparts (Verhagen et al., 2022), but also exhibit a higher risk of developing AD if they are older than 65 years (Wang et al., 2012). Pre-diabetic females, on the other hand, show an earlier onset of dementia and a faster decline in executive function compared to normoglycemic females (Sundermann et al., 2021). The present study identified higher lrFCD in pre-diabetic than in normoglycemic females and no change in males. These functional anomalies affected the precuneus, a cortical region that, in addition to being affected by early AD pathology (Palmqvist et al., 2017), exhibits the greatest sex difference during normal aging, with lrFCD being higher in females than in males (Tomasi and Volkow, 2012). The aberrant increase in functional integrative capacity of precuneus shown by pre-diabetic females in the present study may reflect the activation of a compensatory mechanism aimed at maintaining the function of this cortical hub at the cost of a loss of energy efficiency. We speculate that the drop of sex hormones that typically affects postmenopausal women could favor mitochondrial dysregulation and eventually activate a compensatory increase in oxidative phosphorylation in the precuneus to counteract the increased demand for energy (Demetrius et al., 2021). Supporting this interpretation, the anomalous increase in the number of functional connections of the precuneus was linked to better planning abilities, as revealed by the ToL scores. This is coherent with the role of the precuneus in the neural network responsible for overall planning and planning complexity evaluated through the ToL test (Nitschke et al., 2017). Although it is possible that this compensatory response may delay the progression of cognitive impairment when glucose homeostasis starts to be disrupted, it could be detrimental in the long term (Merlo et al., 2019), which would explain the more rapid deterioration of executive function and earlier onset of AD in pre-diabetic females with MCI (Sundermann et al., 2021). Beyond the plausibility of this hypothesis, we must be cautious when interpreting these results because it is possible that some women in our sample were in the perimenopausal period and others were under hormone replacement therapy.

This study was based on the hypothesis that insulin resistance could act as a moderator of abnormalities in functional brain networks associated with pre-diabetes. For instance, previous studies have reported aberrant patterns of brain functional connectivity related to insulin resistance in patients with type 2 diabetes (Chen et al., 2014; Xia et al., 2015). In line with this hypothesis, we found a negative correlation between HOMA and srFCD in the left mOFC of pre-diabetic individuals, regardless of sex and other demographic and cardiometabolic risk factors. It is important to emphasize that the mOFC, although highly energy efficient (Tomasi et al., 2013), exhibits hypometabolism in pre-diabetic and diabetic patients (Baker et al., 2011). While the reduction of srFCD in this prefrontal hub could result from higher amyloid-beta (Aβ) deposition in this core region of the default mode network (Palmqvist et al., 2017), it could also serve to reduce energy cost and thus maintain the efficiency of functional brain networks linked to this frontal hub. This early metabolic compensation may further contribute to reducing neuronal dysfunctions that ultimately lead to poorer cognition. In line with this hypothesis, the reduction of srFCD in the mOFC correlated with higher MMSE scores, at least in pre-diabetic women.

This prefrontal hub not only showed decreased srFCD with increasing insulin resistance in the pre-diabetic group, but also showed decreased FC with the left posterior cingulate cortex and increased FC with the right parahipocampal gyrus in pre-diabetic individuals. While these FC anomalies with regions of the default mode network were not associated with changes in cognitive function, they are reminiscent of the altered rs-FC patterns observed in middle-aged and older adults with diabetes, which were indeed accompanied by lower scores on different cognitive tests (Hoogenboom et al., 2014; Cui et al., 2015; Guo et al., 2021). The lack of relationship with cognition may be due to the fact that glucose dysregulation and insulin resistance were not sufficiently severe, or that our neuropsychological battery did not include tests sensitive enough to detect such impairment.

Although most of the abnormalities observed in the left mOFC were independent of sex, this prefrontal region showed weaker FC with the right inferior frontal and superior temporal gyri only in pre-diabetic men, which again showed no relationship with cognition, probably for the same reasons mentioned above. These anomalous FC patterns with other regions of the default mode network observed in pre-diabetic men resemble the altered FC patterns shown by diabetic patients (Musen et al., 2012). While highly speculative, it is possible that sex differences in the integrity of brain structure and neurotransmission may have facilitated certain abnormalities in the functional connectivity of the left mOFC to manifest only in pre-diabetic men. In favor of the first hypothesis, a study conducted on more than 2,000 adult brains found that women had greater gray matter volume in the mOFC than men (Lotze et al., 2019). Whether these differences are magnified as a result of metabolic alterations remains to be determined. Previous studies have linked anterior cingulate cortex atrophy to insulin sensitivity measured through the quantitative insulin-sensitivity check index (Castro et al., 2016). Unfortunately, that study did not evaluate the effects of sex so it is unknown whether mOFC integrity is differentially affected by insulin resistance associated with glucose dysregulation in males and females. To date, this relationship has only been evidenced in adolescent females (Gabay et al., 2022). Regarding neurotransmission, there is evidence that concentrations of gamma-aminobutyric acid (GABA) are elevated in the mOFC but not in the precuneus of patients with type 2 diabetes (Thielen et al., 2019). Although it is not known whether this increase in GABA occurs equally in pre-diabetes and whether it affects men more than women, post-mortem examination of human brains has revealed that compared to women, men of all ages show higher concentrations of ionotropic GABA_A_ receptors in the superior temporal gyrus (Pandya et al., 2019), one of the regions with which the left mOFC showed weaker connectivity in pre-diabetic men than in normoglycemic men in the present study.

Our study is subject to several limitations that warrant further consideration. Firstly, and due to the cross-sectional nature of the study, a cause-effect relationship cannot be established. Secondly, our results can only be extended to pre-diabetic middle-aged and older adults with abnormal fasting serum glucose because we did not use the 2-h postprandial glucose tolerance test. Neither did we know whether cortical regions that showed abnormal FCD associated with pre-diabetes had increased Aβ load and/or altered glucose metabolism. Finally, the results observed in females should be interpreted with caution since no hormonal evaluations were performed and no information was collected on reproductive history or hormone replacement therapy. All these aspects should be specifically addressed in future studies.

In conclusion, the results of the present study suggest that early glucose dysregulation in middle-aged and older females affects the functional organization of cortical networks highly vulnerable to AD. These alterations seem to be exacerbated by insulin resistance in pre-diabetic individuals. These findings lead to the hypothesis that, in the short term, lifestyle interventions targeted to cognitively unimpaired older adults with pre-diabetes could contribute to activate compensatory mechanisms aimed to maintain the efficiency of cortical networks and thus hindering the appearance of cellular events that can lead to cognitive impairment, especially in women. Future studies should determine whether sustained activation of these mechanisms over time helps to slow cognitive decline and AD or, on the contrary, may contribute to increase such risk.

## Data availability statement

The original contributions presented in this study are included in the article, further inquiries can be directed to the corresponding author.

## Ethics statement

The studies involving human participants were reviewed and approved by Ethical Committee for Clinical Research of the Junta de Andalucía. The patients/participants provided their written informed consent to participate in this study.

## Author contributions

MA conceived the study and wrote the first draft and is guarantor of this work. JC and MA contributed to the recruitment of participants. All authors contributed to data analysis, data interpretation, and discussion of results, revised the article for important intellectual content, and approved the final version of the manuscript.

## Footnotes

1
https://surfer.nmr.mgh.harvard.edu/

2
https://afni.nimh.nih.gov/afni

3
https://jasp-stats.org/

4
https://www.math.mcgill.ca/keith/surfstat/
